# Mendelian randomization study of the effect of coronary artery calcification on atherosclerotic cardiovascular diseases

**DOI:** 10.1038/s41598-022-19180-x

**Published:** 2022-09-01

**Authors:** Wichanon Sae-jie, Tarinee Tangcharoen, Prin Vathesatogkit, Wichai Aekplakorn, Pimphen Charoen

**Affiliations:** 1 Department of Mathematics, Faculty of Science, Mahidol University, Bangkok, Thailand; 2 Department of Internal Medicine, Faculty of Medicine, Ramathibodi Hospital of Mahidol University, Bangkok, Thailand; 3 Department of Internal Medicine, Faculty of Medicine, Ramathibodi Hospital of Mahidol University, Bangkok, Thailand; 4 Department of Community Medicine, Faculty of Medicine, Ramathibodi Hospital of Mahidol University, Bangkok, Thailand; 5 Department of Tropical Hygiene, Faculty of Tropical Medicine, Mahidol University, Bangkok, Thailand; 6 Integrative Computational Bioscience (ICBS) Center, Mahidol University, Bangkok, Thailand

**Keywords:** Genetic association study, Cardiology

## Abstract

Calcium calcification in the wall of arteries (CAC) leads to a higher risk of atherosclerosis related outcomes, especially myocardial infarction (MI). Nevertheless, the causal role of CAC on other related outcomes is unclear. In this study, we used Mendelian randomization (MR) to systematically investigate the causal role of CAC across a broad range of atherosclerotic cardiovascular diseases including coronary heart disease, angina, MI, ischemic heart disease, stroke, and peripheral vascular disease. Publicly available data from the UK biobank and other data sources were used. Using the two-sample Mendelian randomization (MR) approach, we applied 3 MR models including the inverse variance weighted, the weighted-median, and the weighted-mode methods. Eight SNPs associated with CAC were selected as instrumental variables. We observed causal evidence of CAC on MI consistently across all MR models (P_IVW_ = 1.0 × 10^−4^, P_W-Median_ = 1.1 × 10^−4^, P_W-Mode_ = 3.8 × 10^−2^) and this causation is shown in an acute transmural MI of inferior wall (P_IVW_ = 1.5 × 10^−4^, P_W-Median_ = 4.8 × 10^−5^, P_W-Mode_ = 3.2 × 10^−2^) but not consistently observed in an anterior wall. As each site of acute MI was suggested to have relatively specific mechanisms, our finding suggested that the causal role of CAC on MI is in an inferior wall possibly as a consequence of large calcification from a prolonged process, whereas non-calcified artery plaque or other underlying mechanisms may predominantly play role in an anterior infarction during an advanced atherosclerotic process.

## Introduction

Coronary artery calcification (CAC) is a calcium build up within the walls of arteries which is known as a cumulative lifetime measure of atherosclerosis. CAC occurs very early in the process of atherosclerosis and it can later lead to other atherosclerotic cardiovascular diseases (ASCVD). People with elevated CAC are at the higher risk of overall atherosclerosis related outcomes especially in heart diseases including myocardial infarction (MI)^1^.

Although it has been shown that CAC can be used as a predictor of atherosclerosis, low levels of CAC have also been shown in a number of patients diagnosed with atherosclerosis related diseases^2^. In addition, compared to age-matched controls among physically fit patients, some patients with higher CAC did not show an increased risk of cardiovascular morbidity and mortality^1^. In addition, not only calcified plaque but also non-calcified plaque plays a role in cardiovascular events. Therefore, the role of CAC is unclear and perhaps it is possible that there is a variation in a causal role of CAC on related cardiovascular events by coronary artery location.

Here we applied Mendelian randomization (MR) to investigate the causal role of CAC on a broad range of ASCVD including coronary heart disease (CHD), angina, MI, ischemic heart disease (IHD), stroke, and peripheral vascular disease (PVD). We further looked into their subtypes to thoroughly investigate in more detail. Publicly available data from the UK biobank and other data sources were used to ensure the evidence of causality that we observe.

## Methods

Mendelian randomization (MR) is a method that uses genetic variants to distinguish correlation from causation in observational data^3^. By analogy to the concept of randomized control trials, MR uses genetic variants as a natural experiment and thus less likely to be affected by confounding or reverse causation than conventional observational studies. It provides estimates of the effects of the risk factor over a lifetime.

A key principle of MR is that genetic variants which alter the level of risk factors that itself alters disease risk should be related to disease risk to the extent predicted by their influence on exposure to the risk factor^4^. MR depends on three key assumptions. Firstly, the genetic instrument is (strongly) associated with the risk factor. Secondly, the genetic instrument is independent of observed and unobserved confounders of the risk factor-outcome association. Lastly, the genetic instrument is conditionally independent of the outcome, i.e., no pleiotropy.

### Research design and methods

#### Two-sample mendelian randomization

We applied a two-sample MR approach to incorporate publicly available data from two different genome-wide association study (GWAS) samples, such that, one for the risk factor and the other for the outcome. This framework allows substantial increase in statistical power^5^. Under the two-sample MR approach, we investigate the causal effect of CAC on ASCVD using different MR methods to ensure the result consistency under different conditions, i.e., the inverse variance-weighted method (IVW), the weighted median method (W-Median), the weighted mode method (W-Mode), and MR-Egger regression when there is evidence of pleiotropy. When all genetic variants are valid instruments, IVW is the most efficient estimate of the causal effect. For the weaker assumptions, the median-based method only requires at least half of the variants to be valid instruments, the mode-based method requires the most common causal effect to be consistent with the true causal effect but also allows the remaining instruments to be invalid, and MR-Egger allows genetic variants to have directional pleiotropic effects^6^. We used Cochran Q statistic to assess the presence of heterogeneity^7^ and intercepts of MR-Egger regressions to assess the presence of directional pleiotropy.

In practice, the standard IVW is generally used as the primary analysis method because it is the most efficient analysis method with valid instrumental variables. If a causal effect is detected using IVW method, then other methods will be applied as sensitivity analyses to assess the robustness of the finding^8^. Pseudo r^2^ representing proportion of variance of liability explained by SNPs and F-statistics were calculated to evaluate the strength of instruments.

A nominal significant threshold of *P* < 0.05 and a stringent significant threshold of *P* < 0.008 after a Bonferroni correction in relation to 6 main outcomes (α = 0.05/6 outcomes) were reported. All reported odds ratios (OR) are expressed per one unit increase increment of CAC score. MR analyses were performed using the TwoSampleMR package^9^ in R 4.1.2 software.

### Instrumental variable selection

Eight SNPs with strong and suggestive associations reported in O'Donnell et al., 2011 were selected as instrumental variables (Table 1). In O'Donnell et al., 2011, a meta-analysis of genome-wide association studies for CAC was carried out in 9961 men and women from five independent community-based cohorts, with replication in three additional independent cohorts (n = 6032). In this study, Computed Tomography (CT) was used to assess the quantity of CAC and the Agatston method was used to quantify CAC scores.

**Table 1:** Eight instrumental SNPs and their association summary reported at the discovery stage^15^.

SNPs	Chr	Closest reference gene*	Beta coefficient	SE	*P* value (Discovery stage)	Allele frequency
Association with *P* < 5.00 × 10^−8^						
rs1333049	9	(CDKN2B)	0.269	0.03	7.58 × 10^−19^	0.47
rs9349379	6	PHACTR1	0.211	0.032	2.65 × 10^−11^	0.59
Association with 5.00 × 10^−8^ < *P* < 5.00 × 10^−6^						
rs2026458	6	PHACTR1	0.162	0.031	1.78 × 10^−7^	0.46
rs3809346	13	COL4A2	0.154	0.032	1.25 × 10^−6^	0.43
rs6783981	3	SERPINI1	0.14	0.03	3.94 × 10^−6^	0.51
rs17676451	12	HAL	0.17	0.037	4.08 × 10^−6^	0.22
rs6604023	1	(CDC7)	0.184	0.04	4.29 × 10^−6^	0.18
rs8001186	13	(IRS2)	0.148	0.032	4.51 × 10^−6^	0.67

*SNPs* single-nucleotide polymorphisms; *Chr* chromosome; *SE* standard error; Sample size = 9961.

*Genes for SNPs that are outside the transcript boundary of the protein-coding gene are shown in parentheses [e.g., (CDKN2B)].

Two most strongly associated SNPs at Genome-wide significance level (*P* < 5.0 × 10^−8^) were rs1333049 (*P* = 7.58 × 10^−19^) on the chromosome 9p21 near CDKN2A and CDKN2B, and rs9349379 (*P* = 2.65 × 10^−11^) on chromosome 6p24 within the PHACTR1 gene. We also included 6 SNPs with suggestive association which were rs2026458 (*P* = 1.78 × 10^−7^), rs3809346 (*P* = 9.09 × 10^−6^), rs6783981 (*P* = 3.94 × 10^−6^), rs17676451 (*P* = 4.08 × 10^−6^), rs6604023 (*P* = 4.29 × 10^−6^), and rs8001186 (*P* = 4.51 × 10^−6^) near genes PHACTR1, COL4A2, SERPINI, HAL, CDC7, IRS2, respectively.

### Quality control

If any of our instrumental SNPs was not available in GWAS, available SNP with strong linkage disequilibrium (LD) was used as a proxy SNP (r^2^ > 0.8). For those instrumental SNPs that cannot be certain about their forward strand in the process of data harmonization, they are referred to as palindromic SNPs and they were to be removed from our analysis due to uncertainty of effect harmonization in the two-sample MR.

### Data sources

For the main types of ASCVD, publicly available data from the UK biobank which covered the outcomes of our interest was mainly used. The details of demographics and GWAS analyses for ASCVD outcomes in the UK Biobank data were provided in Supplementary Tables S1 and S2. The UK Biobank has ethical approval by the North West Multi-centre Research Ethics Committee (MREC) for collecting and distributing the data from the participants which covered the work in this study. All methods were carried out in accordance with relevant guidelines and the study protocol conforms to the ethical guidelines of the Declaration of Helsinki (1975). In addition, we searched for other publicly available large datasets (N > 100,000) with complete GWAS summary statistics using both IEU website (https://gwas.mrcieu.ac.uk/) and GWAS catalog (https://www.ebi.ac.uk/gwas/) reported up until June 2021. For subtypes of ASCVD, the UK biobank was used as there was no other large dataset with complete GWAS summary statistics available.

## Results

**Figure 1:**
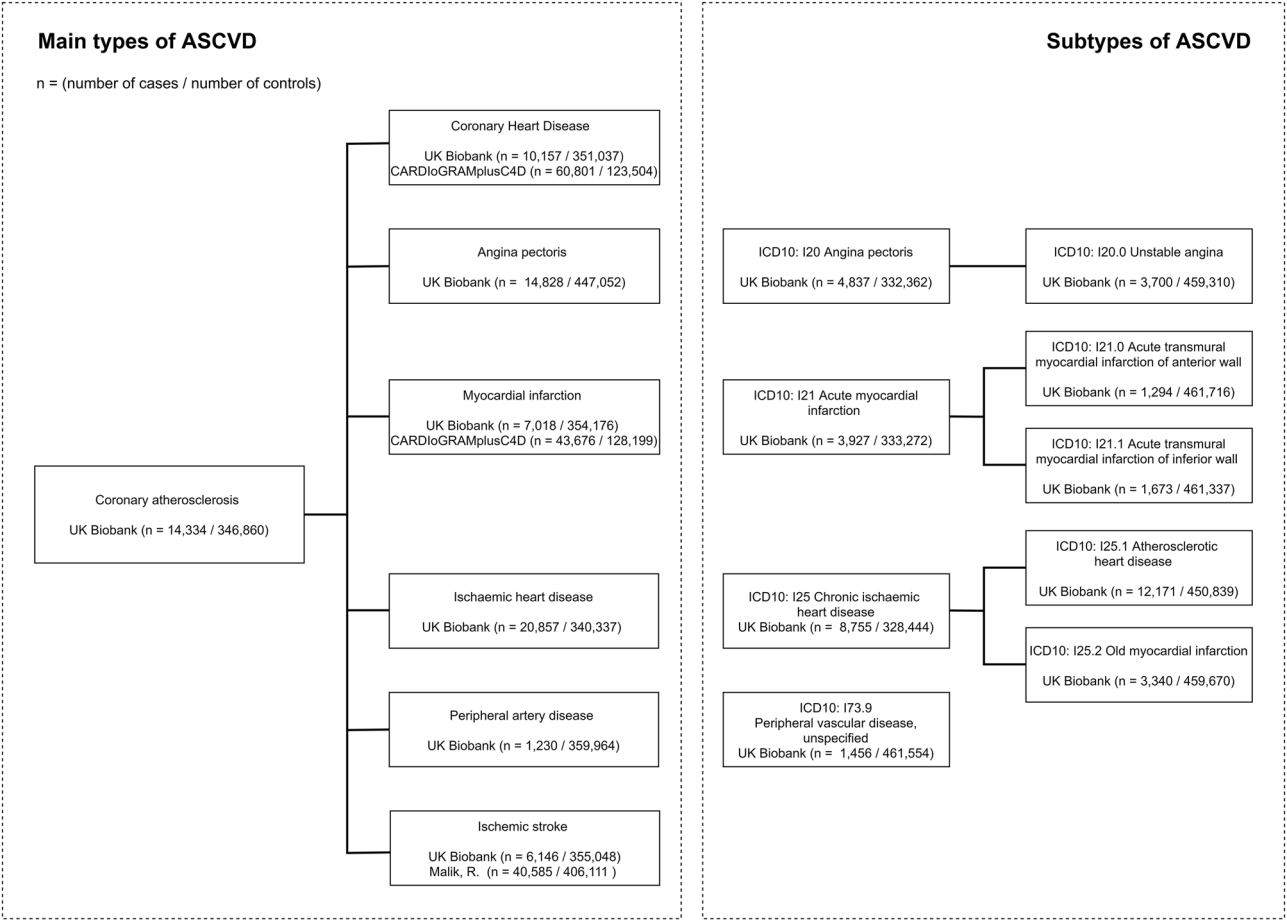
The flow chart of ASCVD outcomes and sources of database.

A summary of data sources used in analyses for both main types and subtypes of ASCVD are shown in Fig. 1. For the main types, publicly available data from the UK biobank which well covered phenotypes of our interest was mainly used. Main types of ASCVD include CHD, angina, MI, IHD, ischemic stroke, and PMD. In addition, we performed parallel analyses on other available large datasets when exist, including MI and CHD reported in the Coronary ARtery DIsease Genome-wide Replication and Meta-analysis plus the Coronary Artery Disease Genetics consortium (CARDIoGRAMplusC4D), and ischemic stroke reported in Malik et al. 2018 (Supplementary Table S3).

For the subtypes of ASCVD, limited GWAS summary statistics are available. Only data reaching our criteria (N > 100,000 and N case > 1000) was obtained from the UK biobank (Supplementary Table S4). In the UK biobank, subtypes were systematically classified using the categories of the International Classification of Diseases External 10th Revision (ICD-10). Subtypes included in our analyses are 1) angina pectoris (I20): unstable angina (I20.0), 2) acute MI (I21): acute transmural MI of anterior wall (I21.0), and acute transmural MI of inferior wall (I21.1), 3) Chronic IHD (I25): atherosclerotic heart disease (I25.1) and old MI (I25.2), and 4) peripheral vascular diseases: other peripheral vascular diseases (I73.9).

Considering the average pleiotropic effect across all genetic variants using intercepts of MR-Egger regressions, we did not observe any evidence of directional pleiotropy across all MRs (*P*-value (Pleiotropy) > 0.05; Tables S5, S6). Using 8 instrumental SNPs for CAC, pseudo r^2^ value was 0.2 and F-statistic was 30, which suggests strong instruments for CAC in this study. Two instrumental SNPs, rs6604023 and rs17676451, were not shown in the GWAS across many phenotypes in the UK biobank data and their proxy SNPs cannot be identified therefore most of our analyses were based on the maximum of 6 instrumental SNPs.

### MR results in the main types of ASCVD

**Figure 2:**
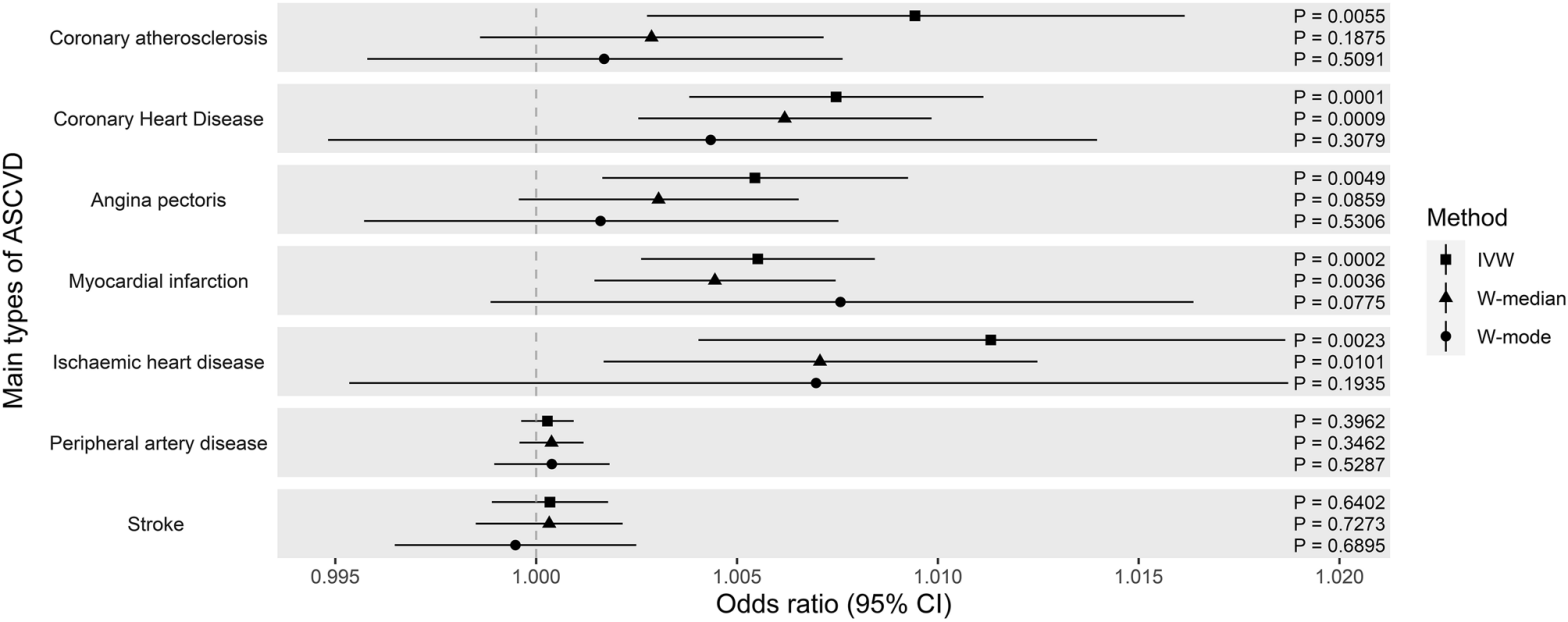
Causal estimates of CAC on main types of ASCVD outcomes (odds ratio with 95% confidence interval) using the UK Biobank data.

Using the UK Biobank, we observed consistent evidence of causality between CAC and MI (IVW: P_IVW_ = 1.9 × 10^−4^, odds ratio by IVW [OR_IVW_] = 1.006; W-Median: P_W-Median_ = 3.6 × 10^−3^, OR_W-Median_ = 1.004, Supplementary Table S5) at a Bonferroni corrected *P*-value < 0.008 in both IVW and W-median (Fig. 2). While this causal evidence did not reach a nominal threshold in the W-mode, some suggestive evidence can be observed (P_W-Mode_ = 7.8 × 10^−2^, OR_W-Mode_ = 1.008). Evidence of causality between CAC and CHD was also shown in both IVW and W-median however this was not the case in the W-mode (P_IVW_ = 6.0 × 10^−5^, OR_IVW_ = 1.008; P_W-Median_ = 8.6 × 10^−4^, OR_W-Median_ = 1.006; P_W-Mode_ = 0.31, OR_W-Mode_ = 1.004). An increased risk of CHD and MI was observed at the greater level of CAC which is concordant with observational studies previously conducted^1,10^.

Although, evidence of causality between CAC and other heart related outcomes were shown using the IVW; coronary atherosclerosis (P_IVW_ = 5.5 × 10^−3^), IHD (P_IVW_ = 2.3 × 10^−3^), and angina pectoris (P_IVW_ = 4.9 × 10^−3^), these evidences were not observed using other MR methods (Fig. 2). Outside the heart, we neither observe the causal evidence in stroke (P_IVW_ = 0.64; P_W-Median_ = 0.73, P_W-Mode_ = 0.70) nor peripheral vascular disease (P_IVW_ = 0.40; P_W-Median_ = 0.35; P_W-Mode_ = 0.53). There was no evidence of directional pleiotropy therefore MR-Egger was not applied (Supplementary Table S6).

We additionally performed parallel analyses on other publicly available large datasets when they exist. Using CARDIoGRAMplusC4D^11^, we observed similar results with the UK Biobank where CAC has causal evidence on both MI and CHD in IVW and W-median (Supplementary Fig. S1), i.e. MI (P_IVW_ = 8.7 × 10^−4^, P_W-Median_ = 8.8 × 10^−3^) and CHD (P_IVW_ = 1.5 × 10^−3^, P_W-Median_ = 4.8 × 10^−2^). This evidence was not shown in the W-mode (CHD: P_W-Mode_ = 0.14, MI: P_W-Mode_ = 0.21). Using GWAS summary data of stroke^12^, attenuated effect confirmed no causal evidence of CAC on stroke as previously observed (P_IVW_ = 0.66; P_W-Median_ = 0.35; P_W-Mode_ = 0. 40). Unfortunately, we did not find any other large datasets that reach our criteria for the rest of our phenotypes of interest. Results from these parallel analyses have so far supported what we previously observed from the UK biobank data.

### MR results in the subtypes of ASCVD

**Figure 3:**
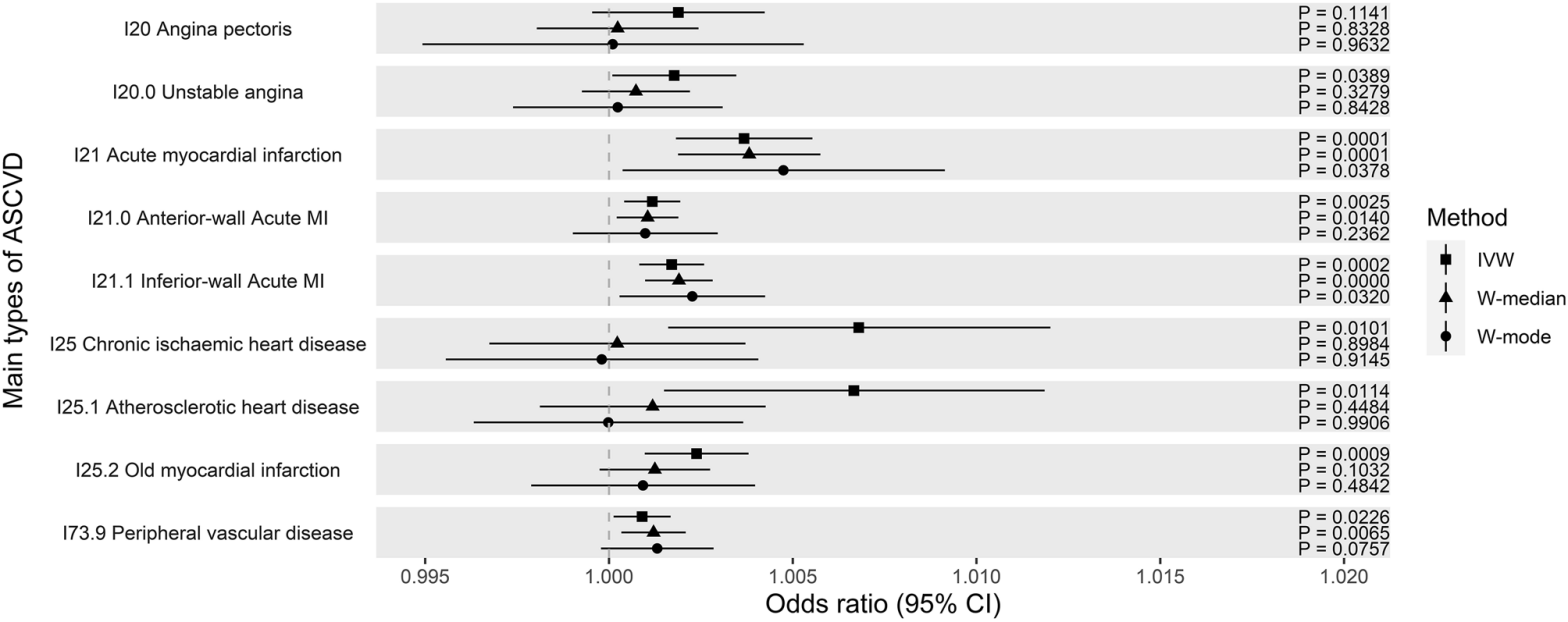
Causal estimates of CAC on subtypes of ASCVD outcomes (odds ratio with 95% confidence interval) using the UK Biobank data.

Using UK biobank data, evidence of causality between CAC and acute MI was consistently observed across all MR methods (P_IVW_ = 1.0 × 10^−4^, OR_IVW_ = 1.001; P_W-Median_ = 1.1 × 10^−4^, OR_W-Median_ = 1.004; P_W-Mode_ = 3.8 × 10^−2^, OR _W-Mode_ = 1.005). When we looked into their subtypes, this causal evidence can only be observed consistently in an acute transmural MI of inferior wall (P_IVW_ = 1.5 × 10^−4^, OR_IVW_ = 1.001; P_W-Median_ = 4.8 × 10^−5^, OR_W-Median_ = 1.002; P_W-Mode_ = 3.2 × 10^−2^, OR _W-Mode_ = 1.002) whereas this was not the case for acute transmural MI of anterior wall (P_IVW_ = 2.5 × 10^−3^, P_W-Median_ = 1.4 × 10^−2^, P_W-Mode_ = 0.24). While the stringent *P*-value can be reached in the IVW and W-median for an inferior wall MI, the nominal level of *P*-value was observed in the W-mode (Fig. 3, Supplementary Fig. S2).

We did not observe further evidence of causality that reached our stringent threshold in angina and IHD subtypes. Only a nominal significance level can be observed under IVW including chronic IHD (P_IVW_ = 1.0 × 10^−2^), and atherosclerotic heart disease (P_IVW_ = 1.1 × 10^−2^) (Fig. 3, Supplementary Table S7). Interestingly, PAD under ICD10 classification also showed some suggestive evidence of causality (P_IVW_ = 6.0 × 10^−2^, P_W-Median_ = 6.5 × 10^−3^, P_W-Mode_ = 7.6 × 10^−2^).

For an acute transmural MI of anterior wall, 5 instrumental SNPs were used to perform MR analyses while the other 3 instrumental SNPs were excluded. Apart from rs6604023 and rs17676451 that cannot identify proxy SNPs across many phenotypes in the UK biobank data, rs1333049 was further excluded due to a potential palindromic SNP. To further check whether the causal evidence observed in an inferior wall MI compared to an anterior wall MI was not influenced by rs1333049, we performed sensitivity analyses when rs1333049 was excluded. The causal evidence in the inferior wall MI was maintained even when remaining 5 instrumental SNPs were included (Supplementary Table S8; P_IVW_ = 9.0 × 10^−4^, P_W-Median_ = 5.0 × 10^−4^, P_W-Mode_ = 3.4 × 10^−2^). Therefore, this assured that the causal evidence previously observed in an inferior wall MI was not due to an absence of the missing instrumental SNP.

## Conclusions

Calcium calcification in the wall of arteries is leading to a higher risk of atherosclerosis related outcomes. Using the MR approach, we showed that CAC has a causal role on MI and this causation is shown in an acute transmural MI of inferior wall but not consistently observed in an anterior wall. It was previously suggested that each site of acute myocardial infarction has relatively specific mechanisms while more investigations are still further required to improve an understanding of these underlying mechanisms. Vascular calcification is an active and regulated process therefore an early detection of CAC on asymptomatic phenomenon is thus important, and our study suggested that delaying progression of CAC could prevent an acute transmural MI in an inferior wall.

## Discussion

An anterior/inferior wall MI occurs when left/right descending coronary arteries lack blood supply. In general, patients with an inferior wall MI usually visit hospitals at a much later stage compared to patients with an anterior wall MI. Due to a prolonged period of time, larger calcifications are often observed in inferior wall MI patients. In addition, when inferior wall MI patients come to hospitals, they often show less related symptoms, e.g., stomach ache, fatigue, and lightheadedness. This agrees with the previous report that patients with larger calcifications are more often asymptomatic^13^. Perhaps our study further strengthens the role of this larger calcification and suggests the causal role of lifetime cumulative of CAC on MI in an inferior wall in particular.

Each site of acute myocardial infarction was suggested to have relatively specific mechanisms. The predominant pathophysiology in an anterior infarction was reported as an advanced atherosclerotic process^14^. Perhaps, it may be non-calcified artery plaque or other underlying mechanisms that mainly contribute to an infarction in an anterior wall during this advanced atherosclerosis process. In addition, our study used instrumental SNPs of CAC score which quantify the level of calcification detected by CT. As CT mainly detects macrocalcification, any consequence from microcalcification may not well be captured in our study.

While we consistently observed causal evidence of CAC on an inferior wall MI across 3 MR methods, only the result from IVW method from an anterior wall MI passed our strict threshold but not in other sensitivity analyses. Because the effect size of CAC on MI was shown to be small, further investigation using larger data will be required to confirm an absence of causality between CAC and an acute transmural MI of anterior wall.

While the significant threshold of *P*-value = 0.008 under the Bonferroni correction can be considered to be too strict because those 6 main types of ASCVD are unlikely to be independent, the significant threshold of *P*-value = 0.05 could also be too relaxed. In this paper, we reported both significant thresholds while an appropriate threshold would lie in between.

For the validity of instrumental SNPs, we included both SNPs with strong association (*P* < 5 × 10^−8^) and those with suggestive associations (5 × 10^−8^ < *P* < 5 × 10^−6^) from GWAS. While two of the strong associated SNPs (rs1333049 near CDKN2B, and rs9349379 in PHACTR1), and two of the suggestive SNPs (rs2026458 in PHACTR1, and rs3809346 in COL4A2) were previously replicated^15^, little is known about the other 4 remaining SNPs where their biological mechanisms related to CAC are unclear. In two-sample MR, this could also lead to bias from weak instruments which shifts the MR estimate towards the direction of the null^3^. Perhaps this may also explain weaker associations between CAC and MI as well as CAC and an inferior wall MI observed in the W-mode which assumes the most common causal effect to be consistent with the true causal effect.

It was previously shown that CAC is an independent predictor of stroke^16^; however, our study did not find the causal relationship. For PAD, we observed some suggestive evidence of causality when patients were classified under ICD10 classification. However, this was shown at the nominal significance level and inconsistent results were observed across a range of our analyses. It is known that an intimal calcification is associated with coronary artery disease while a medial calcification mostly affects the peripheral arteries of the lower extremities which is routinely observed in patients with PAD^17^. Perhaps, macrocalcification detected by computed tomography (CT) CAC score in our study may not be the best measurement of medial calcification to investigate its causal role on PAD, and alternative scoring systems may require, e.g., peripheral arterial calcium scoring^17^ and lower limb arterial calcification scoring^18^. More research on the causal role of different types of calcifications on PAD should be further investigated based on our suggestive evidence.

While underlying mechanisms of each site of acute myocardial infarction are an on-going work^13^, our study observed the causation between CAC and an acute transmural MI of inferior wall but not in an anterior wall. The causal role of lifetime cumulative of CAC on an inferior wall MI suggested in our study should be further investigated. Due to a small effect size observed, larger sample size should be further explored to confirm the causal evidence and validate the causal estimate in our study.

## Supplementary Information

Supplementary Information.
