# Supplementary material for: Mendelian randomization study of the effect of coronary artery calcification on atherosclerotic cardiovascular diseases
Source: Sci Rep. 2022 Sep 1;12:14829. doi: 10.1038/s41598-022-19180-x (PMC9437097; doi:10.1038/s41598-022-19180-x)
Supplement: Supplementary file 1 — Supplementary Information. [file 41598_2022_19180_MOESM1_ESM.docx]

## Supplementary Information

[**Table S1** The demographics of 503,310 participants in the UK Biobank data collected between 2006 to 2010. The participants who lived throughout England, Wales, and Scotland were recruited into UK Biobank (Ref). More details on the baseline assessments are available in this repository (https://www.ukbiobank.ac.uk/enable-your-research/about-our-data/baseline-assessment) 2](#_Toc108445343)

[**Table S2** The description of GWAS summary datasets and GWAS method obtained from UK Biobank using 3 of 18 batches in https://gwas.mrcieu.ac.uk/ 3](#_Toc108445344)

[**Table S3** Information of data sources for the broad range of the related ASCVD events in UK Biobank and consortia 4](#_Toc108445345)

[**Table S4** Information of data sources for subtypes of ASCVD from UK Biobank 4](#_Toc108445346)

[Table S5 Associations (odds ratio per 1 standard deviation increase in cardiovascular conditions and 95% confidence interval) between CAC with a broad range of the related ASCVD events included studies and consortia. All estimations were based on the inverse variance weighted method, weighted median, and weighted mode. 5](#_Toc108445347)

[**Table S6** Associations (odds ratio per 1 standard deviation increase in cardiovascular conditions and 95% confidence interval) between CAC with a broad range of related ASCVD events from UK Biobank and consortia. Pleiotropy was tested by the MR-Egger intercept and heterogeneity was tested by Cochran’s Q statistic. 6](#_Toc108445348)

[**Table S7** Associations (odds ratio per 1 standard deviation increase in cardiovascular conditions and 95% confidence interval) between CAC with subtypes of the related ASCVD events from UK Biobank. All estimations were based on the inverse variance weighted method, weighted median, and weighted mode. 7](#_Toc108445349)

[**Table S8** Associations (o dds ratio per 1 standard deviation increase in cardiovascular conditions and 95% confidence interval) between CAC with ICD10: I21.1 Acute transmural myocardial infarction of inferior wall using 5 instrumental SNPs 8](#_Toc108445350)

[**Table S9** Associations (odds ratio per 1 standard deviation increase in cardiovascular conditions and 95% confidence interval) between CAC with subtypes of the related ASCVD events from UK Biobank. Pleiotropy was tested by the MR-Egger intercept and heterogeneity was tested by the Cochran’s Q statistic. 8](#_Toc108445351)

**Table S1** The demographics of 503,310 participants in the UK Biobank data collected between 2006 to 2010. The participants who lived throughout England, Wales, and Scotland were recruited into UK Biobank^1^. More details on the baseline assessments are available in this repository (https://www.ukbiobank.ac.uk/enable-your-research/about-our-data/baseline-assessment)

| **Sex and Characteristic** | **Age 45–54 Years** | | **Age 55–64 Years** | |
| --- | --- | --- | --- | --- |
|  | **No. of Persons** | **Mean (SD) or %** | **No. of Persons** | **Mean (SD) or %** |
| **Men** (n = 229,486, 45.6%) | | | | |
| BMI | 61,860 | 27.8 (4.4) | 94,776 | 27.9 (4.3) |
| Weight, kg | 61,929 | 86.9 (15.1) | 94,875 | 86.0 (14.3) |
| Height, cm | 61,919 | 176.5 (6.9) | 94,901 | 175.4 (6.7) |
| WC, cm | 62,010 | 96.1 (11.5) | 95,031 | 97.7 (11.4) |
| **Prevalence (%) of related ASCVD events** | | | | |
| Cardiovascular disease^a^ | - | 4.6 | - | 11.5 |
| Ischemic heart disease^b^ | - | 2.8 | - | 7.9 |
| Stroke | - | 0.8 | - | 1.9 |
| Angina | - | 1.8 | - | 5.3 |
| Myocardial infarction | - | 1.7 | - | 4.5 |
| Abnormal heart rhythm | - | 1.5 | - | 3.1 |
| **Women**(n = 273,824, 54.4%) |  |  |  |  |
| BMI | 79,714 | 26.9 (5.4) | 116,303 | 27.3 (5.1) |
| Weight, kg | 79,738 | 71.8 (14.8) | 116,344 | 71.6 (13.8) |
| Height, cm | 79,792 | 163.4 (6.3) | 116,429 | 162.0 (6.2) |
| WC, cm | 79,809 | 83.6 (12.8) | 116,471 | 85.5 (12.5) |
| **Prevalence (%) of related ASCVD events** | |  |  |  |
| Cardiovascular disease^a^ | - | 2.4 | - | 5.0 |
| Ischemic heart disease^b^ | - | 0.9 | - | 2.6 |
| Stroke | - | 0.6 | - | 1.0 |
| Angina | - | 0.7 | - | 2.1 |
| Myocardial infarction | - | 0.3 | - | 0.9 |
| Abnormal heart rhythm | - | 1.4 | - | 2.2 |
| Abbreviations: BMI, body mass index; SD, standard deviation; WC waist circumference; ASCVD, atherosclerotic cardiovascular disease | | | | |
| ^a^ Cardiovascular disease included angina, heart attack, stroke, heart murmur, and irregular heart rhythm. | | | | |
| ^b^ Ischemic heart disease included heart attack or angina. | | | | |

**Table S2** The description of GWAS summary datasets and GWAS method obtained from UK Biobank using 3 of 18 batches in <https://gwas.mrcieu.ac.uk/>

| **Batch** | **Software** | **Method for binary outcomes** | **Covariates** | **Details** |
| --- | --- | --- | --- | --- |
| uk-a | SNPTEST | Bayesian case-control test | Sex, age, genotype array, and the first 10 PCs | Neale lab analysis from round 1^2^ |
| uk-b | PLINKv2.00 | Linear/logistic regression | Sex, genotype array, and the first 10 PCs | IEU analysis^3^ |
| uk-d | PHESANT | Linear/logistic regression | Sex, and the first 10 PCs | Neale lab analysis from round 2^4^ |
| Abbreviations: GWAS, Genome-wide association study; PC, principal component; IEU, Integrative Epidemiology Unit | | | | |

**Table S3** Information of data sources for the broad range of the related ASCVD events in UK Biobank and consortia

| **Disease outcomes** | **UK Biobank** | | | | | **Consortia** | | | | |
| --- | --- | --- | --- | --- | --- | --- | --- | --- | --- | --- |
|  | **ID** | **No. of Cases** | **No. of Control** | **Ethnicity** | **Name of Consortia** | **ID** | **No. of Cases** | **No. of Control** | **Ethnicity** | **Name of Consortia** |
| Coronary atherosclerosis | ukb-d-I9_CORATHER | 14,334 | 346,860 | European | Neale lab^7^ | - | - | - | - | - |
| Coronary Heart Disease | ukb-d-I9_CHD | 10,157 | 351,037 | European | Neale lab^7^ | ieu-a-7 | 60,801 | 123,504 | Mixed | CARDIoGRAMplusC4D^6^ |
| Angina pectoris | ukb-b-8468 | 14,828 | 447,052 | European | MRC-IEU^8^ | - | - | - | - | - |
| Myocardial infarction | ukb-d-I9_MI | 7,018 | 354,176 | European | Neale lab^7^ | ieu-a-798 | 43,676 | 128,199 | Mixed | CARDIoGRAMplusC4D^6^ |
| Ischaemic heart disease, wide definition | ukb-d-I9_IHD | 20,857 | 340,337 | European | Neale lab^7^ | - | - | - | - | - |
| Peripheral artery disease | ukb-d-I9_PAD | 1,230 | 359,964 | European | Neale lab^7^ | - | - | - | - | - |
| Stroke | ukb-d-C_STROKE | 6,146 | 355,048 | European | Neale lab^7^ | ebi-a-GCST005838 | 40,585 | 406,111 | European | (Malik et al., 2018)^5^ |
| Abbreviations: ASCVD, atherosclerotic cardiovascular disease; MRC-IEU, The MRC Integrative Epidemiology Unit at the University of Bristol;  CARDIoGRAMplusC4D, Coronary ARtery DIsease Genome wide Replication and Meta-analysis (CARDIoGRAM) plus The Coronary Artery Disease (C4D) Genetics | | | | | | | | | | |

**Table S4** Information of data sources for subtypes of ASCVD from UK Biobank

| **ICD-10** | **Disease outcomes** | **UK Biobank** | | | | |
| --- | --- | --- | --- | --- | --- | --- |
|  |  | **GWAS ID** | **No. of Cases** | **No. of Control** | **Ethnicity** | **Name of Consortia** |
| I20-I25 | Ischaemic heart diseases |  |  |  |  |  |
| I20 | Angina pectoris | ukb-a-532 | 4,837 | 332,362 | European | Neale Lab^9^ |
| I20.0 | Unstable angina | ukb-b-12047 | 3,700 | 459,310 | European | MRC-IEU^8^ |
| I21 | Acute myocardial infarction | ukb-a-533 | 3,927 | 333,272 | European | Neale Lab^9^ |
| I21.0 | Acute transmural myocardial infarction of anterior wall | ukb-b-453 | 1,294 | 461,716 | European | MRC-IEU^8^ |
| I21.1 | Acute transmural myocardial infarction of inferior wall | ukb-b-5126 | 1,673 | 461,337 | European | MRC-IEU^8^ |
| I25 | Chronic ischaemic heart disease | ukb-a-534 | 8,755 | 328,444 | European | Neale Lab^9^ |
| I25.1 | Atherosclerotic heart disease | ukb-b-1668 | 12,171 | 450,839 | European | MRC-IEU^8^ |
| I25.2 | Old myocardial infarction | ukb-b-16662 | 3,340 | 459,670 | European | MRC-IEU^8^ |
| I73.9 | Peripheral vascular disease, unspecified | ukb-b-4929 | 1,456 | 461,554 | European | MRC-IEU^8^ |
| Abbreviations: ASCVD, atherosclerotic cardiovascular disease; MRC-IEU, The MRC Integrative Epidemiology Unit at the University of Bristol;  ICD-10, the 10th revision of the International Statistical Classification of Diseases and Related Health Problems | | | | | | |

**Table S5** Associations (odds ratio per 1 standard deviation increase in cardiovascular conditions and 95% confidence interval) between CAC with a broad range of the related ASCVD events included studies and consortia. All estimations were based on the inverse variance weighted method, weighted median, and weighted mode.

| **Group 1 Main types of heart disease** | | | | | | |
| --- | --- | --- | --- | --- | --- | --- |
| **Outcome** | **Inverse variance weighted** | | **Weighted median** | | **Weighted mode** | |
|  | **OR (95% CI)** | **P-value** | **OR (95% CI)** | **P-value** | **OR (95% CI)** | **P-value** |
| **UK Biobank** |  |  |  |  |  |  |
| Coronary atherosclerosis | 1.0094(1.0028,1.0161) | 5.53E-03 | 1.0029(0.9986,1.0072) | 1.88E-01 | 1.0017(0.9958,1.0076) | 5.09E-01 |
| Coronary Heart Disease | 1.0075(1.0038,1.0111) | 6.00E-05 | 1.0062(1.0025,1.0098) | 8.63E-04 | 1.0043(0.9948,1.0140) | 3.08E-01 |
| Angina pectoris | 1.0054(1.0016,1.0093) | 4.92E-03 | 1.0030(0.9996,1.0065) | 8.59E-02 | 1.0016(0.9957,1.0075) | 5.31E-01 |
| Myocardial infarction | 1.0055(1.0026,1.0084) | 1.92E-04 | 1.0044(1.0015,1.0075) | 3.61E-03 | 1.0076(0.9989,1.0164) | 7.75E-02 |
| Ischaemic heart disease, wide definition | 1.0113(1.0040,1.0187) | 2.26E-03 | 1.0071(1.0017,1.0125) | 1.01E-02 | 1.0070(0.9953,1.0187) | 1.93E-01 |
| Peripheral artery disease | 1.0003(0.9996,1.0009) | 3.96E-01 | 1.0004(0.9996,1.0012) | 3.46E-01 | 1.0004(0.9990,1.0018) | 5.29E-01 |
| Stroke | 1.0003(0.9989,1.0018) | 6.40E-01 | 1.0003(0.9985,1.0022) | 7.27E-01 | 0.9995(0.9965,1.0025) | 6.90E-01 |
| **Consortia** |  |  |  |  |  |  |
| Coronary heart disease | 1.3509(1.1218,1.6267) | 1.51E-03 | 1.0979(1.0007,1.2045) | 4.82E-02 | 1.0730(0.9699,1.1871) | 1.39E-01 |
| Myocardial infarction | 1.3725(1.1392,1.6536) | 8.67E-04 | 1.1724(1.0409,1.3205) | 8.78E-03 | 1.0980(0.9348,1.2898) | 2.05E-01 |
| Stroke | 0.9889(0.9414,1.0388) | 6.56E-01 | 0.9739(0.9213,1.0295) | 3.50E-01 | 0.9640(0.8907,1.0433) | 3.98E-01 |
| Abbreviations: ASCVD, atherosclerotic cardiovascular disease; OR, odds ratio | | | | | | |

**Table S6** Associations (odds ratio per 1 standard deviation increase in cardiovascular conditions and 95% confidence interval) between CAC with a broad range of related ASCVD events from UK Biobank and consortia. Pleiotropy was tested by the MR-Egger intercept and heterogeneity was tested by Cochran’s Q statistic.

| **Group 2: Subtypes of heart disease** | | | | | | |
| --- | --- | --- | --- | --- | --- | --- |
|  | **MR-Egger** | | | | **Heterogeneity test (IVW)** | |
| **Outcome** | **OR (95% CI)** | **P-value** | **P-value (Pleiotropy)** | **Egger Intercept** | **Q** | **P-value**  **(Q)** |
| ICD10: I20 Angina pectoris | 1.0127(0.9968,1.0290) | 1.80E-01 | 2.38E-01 | -1.82E-03 | 17.274 | 8.33E-03 |
| ICD10: I20.0 Unstable angina | 1.0110(1.0004,1.0218) | 9.73E-02 | 1.45E-01 | -1.55E-03 | 21.865 | 1.28E-03 |
| ICD10: I21 Acute myocardial infarction | 1.0159(1.0057,1.0261) | 2.77E-02 | 6.33E-02 | -2.04E-03 | 13.308 | 3.84E-02 |
| ICD10: I21.0 Acute transmural myocardial infarction of anterior wall | 1.0060(1.0021,1.0099) | 5.81E-02 | 9.34E-02 | -8.02E-04 | 6.546 | 1.62E-01 |
| ICD10: I21.1 Acute transmural myocardial infarction of inferior wall | 1.0063(1.0010,1.0116) | 8.12E-02 | 1.64E-01 | -7.62E-04 | 9.824 | 8.04E-02 |
| ICD10: I25 Chronic ischaemic heart disease | 1.0313(0.9959,1.0679) | 1.44E-01 | 2.31E-01 | -4.07E-03 | 47.390 | 1.56E-08 |
| ICD10: I25.1 Atherosclerotic heart disease | 1.0338(1.0000,1.0687) | 1.07E-01 | 1.74E-01 | -4.49E-03 | 63.915 | 7.18E-12 |
| ICD10: I25.2 Old myocardial infarction | 1.0128(1.0064,1.0193) | 1.12E-02 | 2.35E-02 | -1.75E-03 | 16.967 | 9.41E-03 |
| ICD10: I73.9 Peripheral vascular disease, unspecified | 1.0036(0.9986,1.0087) | 2.51E-01 | 3.60E-01 | -4.54E-04 | 6.026 | 1.97E-01 |
| Abbreviations: ASCVD, atherosclerotic cardiovascular disease; ICD-10, the 10th revision of the International Statistical Classification of Diseases and Related Health Problems; IVW, inverse variance weighted method; OR, odds ratio | | | | | | |

**Table S7** Associations (odds ratio per 1 standard deviation increase in cardiovascular conditions and 95% confidence interval) between CAC with subtypes of the related ASCVD events from UK Biobank. All estimations were based on the inverse variance weighted method, weighted median, and weighted mode.

| **Group 2: Subtypes of heart disease** | | | | | | |
| --- | --- | --- | --- | --- | --- | --- |
| **Outcome (ICD10)** | **Inverse variance weighted** | | **Weighted median** | | **Weighted mode** | |
|  | **OR (95% CI)** | **P-value** | **OR (95% CI)** | **P-value** | **OR (95% CI)** | **P-value** |
| ICD10: I20 Angina pectoris | 1.0019(0.9995,1.0042) | 1.14E-01 | 1.0002(0.9980,1.0024) | 8.33E-01 | 1.0001(0.9949,1.0053) | 9.63E-01 |
| ICD10: I20.0 Unstable angina | 1.0018(1.0001,1.0035) | 3.89E-02 | 1.0007(0.9993,1.0022) | 3.28E-01 | 1.0002(0.9974,1.0031) | 8.43E-01 |
| ICD10: I21 Acute myocardial infarction | 1.0011(0.9998,1.0025) | 1.00E-04 | 1.0038(1.0019,1.0058) | 1.10E-04 | 1.0047(1.0004,1.0091) | 3.78E-02 |
| ICD10: I21.0 Acute transmural myocardial infarction of anterior wall | 1.0037(1.0018,1.0055) | 2.45E-03 | 1.0011(1.0002,1.0019) | 1.40E-02 | 1.0010(0.9990,1.0030) | 2.36E-01 |
| ICD10: I21.1 Acute transmural myocardial infarction of inferior wall | 1.0012(1.0004,1.0019) | 1.50E-04 | 1.0019(1.0010,1.0028) | 4.80E-05 | 1.0023(1.0003,1.0043) | 3.20E-02 |
| ICD10: I25 Chronic ischaemic heart disease | 1.0017(1.0008,1.0026) | 1.01E-02 | 1.0002(0.9967,1.0037) | 8.98E-01 | 0.9998(0.9956,1.0041) | 9.15E-01 |
| ICD10: I25.1 Atherosclerotic heart disease | 1.0014(1.0004,1.0023) | 1.14E-02 | 1.0012(0.9981,1.0043) | 4.48E-01 | 1.0000(0.9963,1.0037) | 9.91E-01 |
| ICD10: I25.2 Old myocardial infarction | 1.0068(1.0016,1.0120) | 9.20E-04 | 1.0012(0.9997,1.0028) | 1.03E-01 | 1.0009(0.9979,1.0040) | 4.84E-01 |
| ICD10: I73.9 Peripheral vascular disease, unspecified | 1.0067(1.0015,1.0119) | 2.26E-02 | 1.0012(1.0003,1.0021) | 6.48E-03 | 1.0013(0.9998,1.0028) | 7.57E-02 |
| Abbreviations: ASCVD, atherosclerotic cardiovascular disease; OR, odds ratio | | | | | | |

**Table S8** Associations (odds ratio per 1 standard deviation increase in cardiovascular conditions and 95% confidence interval) between CAC with ICD10: I21.1 Acute transmural myocardial infarction of inferior wall using 5 instrumental SNPs

| **Sensitivity Analysis: 5 instrumental SNPs** | | | | | | | |
| --- | --- | --- | --- | --- | --- | --- | --- |
| **Outcome (ICD10)** | **No. of SNPs** | **Inverse variance weighted** | | **Weighted median** | | **Weighted mode** | |
|  |  | **OR (95% CI)** | **P-value** | **OR (95% CI)** | **P-value** | **OR (95% CI)** | **P-value** |
| ICD10: I21.1 Acute transmural myocardial infarction of inferior wall | 5 | 1.0018(1.0007,1.0028) | 9.02E-04 | 1.0021(1.0012,1.0031) | 5.02E-04 | 1.0024(1.0009,1.0039) | 3.35E-02 |
| Abbreviations: ASCVD, atherosclerotic cardiovascular disease; ICD-10, the 10th revision of the International Statistical Classification of Diseases and Related Health Problems; OR, odds ratio; SNP, Single-nucleotide polymorphism | | | | | | | |

**Table S9** Associations (odds ratio per 1 standard deviation increase in cardiovascular conditions and 95% confidence interval) between CAC with subtypes of the related ASCVD events from UK Biobank. Pleiotropy was tested by the MR-Egger intercept and heterogeneity was tested by the Cochran’s Q statistic.

| **Group 1: General forms of heart disease** | | | | | | |
| --- | --- | --- | --- | --- | --- | --- |
|  | **MR-Egger** | | | | **Heterogeneity test (IVW)** | |
| **Outcome** | **OR (95% CI)** | **P-value** | **P-value (Pleiotropy)** | **Egger Intercept** | **Q** | **P-value  (Q)** |
| **UK Biobank** |  |  |  |  |  |  |
| Coronary atherosclerosis | 1.042(0.997,1.089) | 1.29E-01 | 2.16E-01 | -5.36E-03 | 57.019 | 1.81E-10 |
| Coronary Heart Disease | 1.026(1.002,1.051) | 9.07E-02 | 1.95E-01 | -3.06E-03 | 23.480 | 6.51E-04 |
| Angina pectoris | 1.025(1.001,1.051) | 1.02E-01 | 1.75E-01 | -3.30E-03 | 28.295 | 8.27E-05 |
| Myocardial infarction | 1.022(1.005,1.040) | 5.66E-02 | 1.20E-01 | -2.81E-03 | 21.237 | 1.66E-03 |
| Ischemic heart disease | 1.044(0.994,1.097) | 1.46E-01 | 2.54E-01 | -5.45E-03 | 47.647 | 1.39E-08 |
| Peripheral artery disease | 1.002(0.998,1.007) | 3.75E-01 | 4.27E-01 | -3.48E-04 | 2.730 | 8.42E-01 |
| Ischemic stroke | 0.995(0.985,1.006) | 3.96E-01 | 3.62E-01 | 8.96E-04 | 2.030 | 9.17E-01 |
| **Consortia** |  |  |  |  |  |  |
| Coronary artery disease | 4.253(1.491,12.126) | 4.24E-02 | 8.25E-02 | -1.95E-01 | 100.022 | 2.48E-19 |
| Myocardial infarction | 4.275(1.487,12.290) | 4.29E-02 | 8.64E-02 | -1.93E-01 | 81.453 | 1.79E-15 |
| Ischemic stroke | 1.021(0.690,1.510) | 9.22E-01 | 8.78E-01 | -5.39E-03 | 8.160 | 2.27E-01 |
| Abbreviations: ASCVD, atherosclerotic cardiovascular disease; IVW, inverse variance weighted method; OR odds ratio | | | | | | |

**Fig. S1** Forest plot of estimated causal effects from three methods using other consortia and study data.


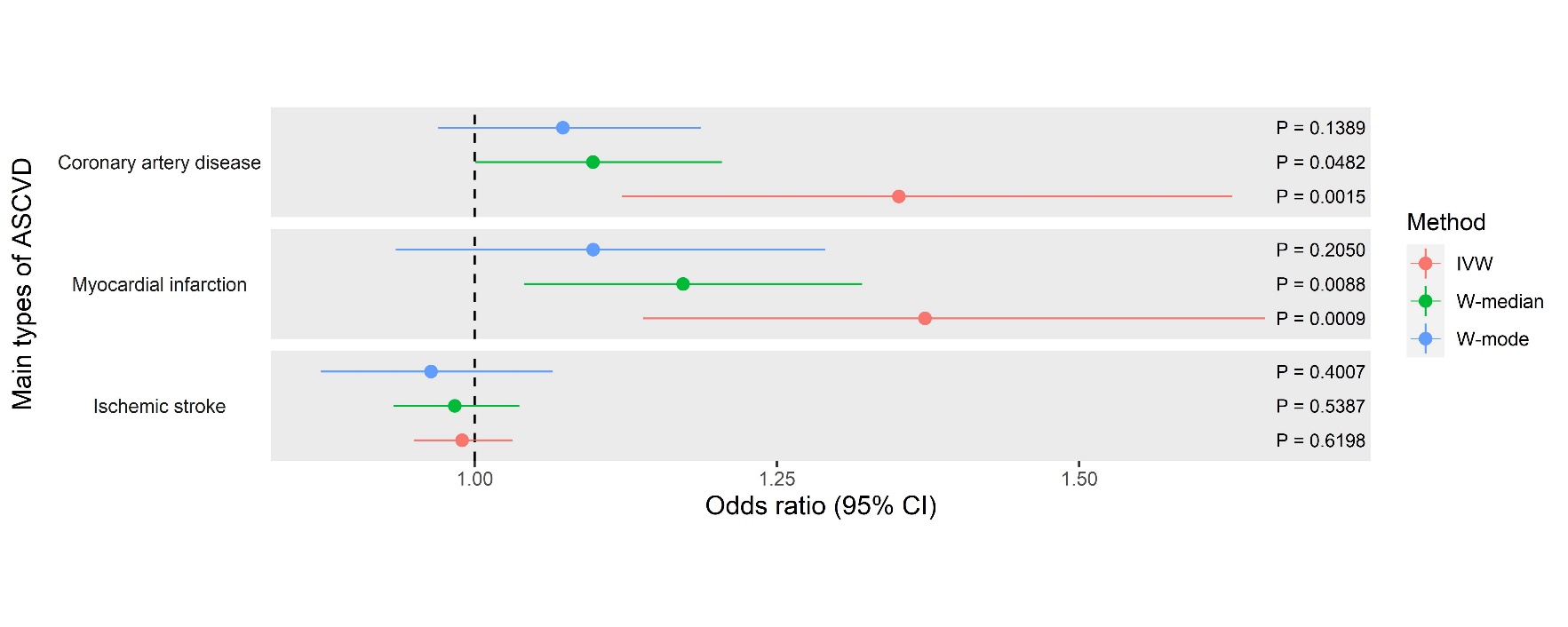


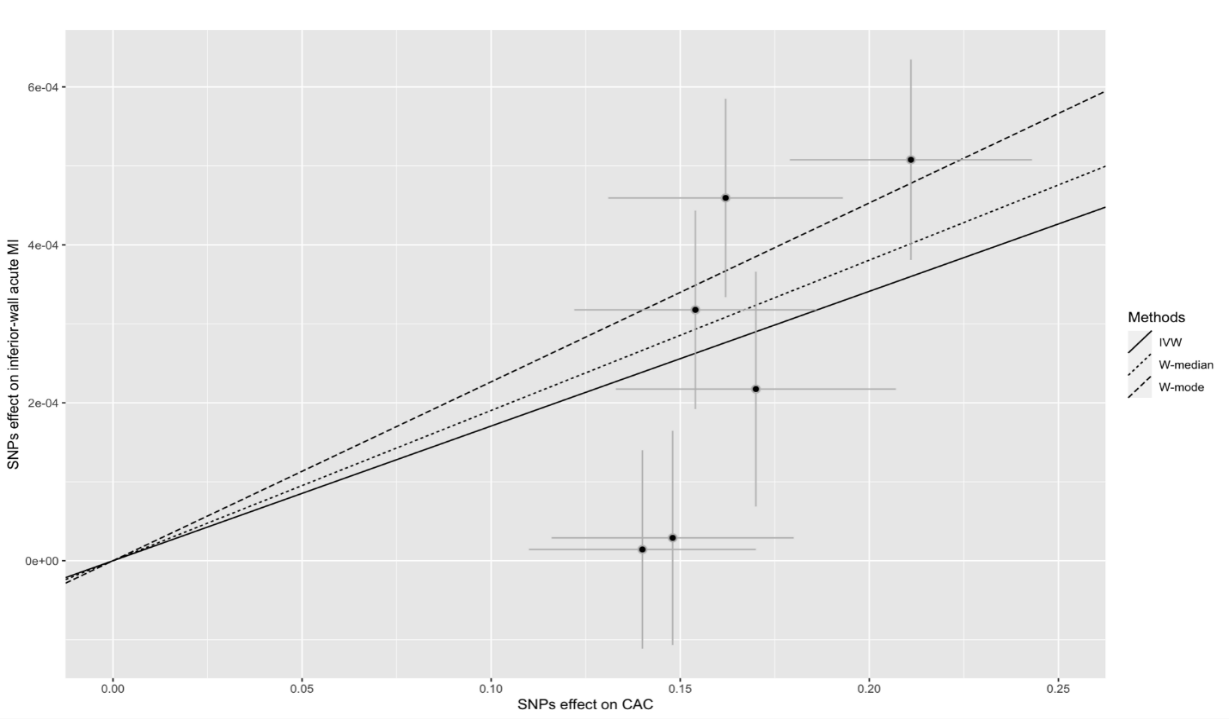
**Fig. S2** Scatter plot of SNP-Inferior wall acute MI associations against SNP-CAC associations with estimates from 3 MR methods

**Supplementary References**

1 Fry A, Littlejohns TJ, Sudlow C, Doherty N, Adamska L, Sprosen T, Collins R, Allen NE. Comparison of Sociodemographic and Health-Related
Characteristics of UK Biobank Participants With Those of the General Population. Am J Epidemiol. 2017 Nov 1;186(9):1026-1034.

2 UK Biobank Phasing and Imputation Documentation Version 1.2 [Internet]. 2015. Available from: https://biobank.ndph.ox.ac.uk/showcase/ukb/docs/impute_ukb_v1.pdf. Accessed 11 July 2022.

3 Mitchell, R. (Creator), Elsworth, B. L. (Creator), Mitchell, R. (Creator), Raistrick, C. A. (Creator), Paternoster, L. (Creator), Hemani, G. (Creator), Gaunt, T. R. (Creator) (19 Feb 2019). MRC IEU UK Biobank GWAS pipeline version 2. University of Bristol. 10.5523/bris.pnoat8cxo0u52p6ynfaekeigi

4 Neale Lab UKB Round 2 GWAS summary statistics [Internet]. 2015. Available from: https://github.com/Nealelab/UK_Biobank_GWAS. Accessed 11
July 2022.

5 Malik R, Chauhan G, Traylor M, Sargurupremraj M, Okada Y, Mishra A, et al. Multiancestry genome-wide association study of 520,000 subjects
identifies 32 loci associated with stroke and stroke subtypes. Nat Genet. 2018;50(4):524-37.

6 Nikpay M, Goel A, Won HH, Hall LM, Willenborg C, Kanoni S, et al. A comprehensive 1,000 Genomes-based genome-wide association meta-analysis of coronary artery disease. Nat Genet. 2015;47(10):1121-30.

7 UK Biobank [Internet]. 2018. Available from: http://www.nealelab.is/uk-biobank/. Accessed 10 May 2021.

8 TR (2019): MRC IEU UK Biobank GWAS pipeline version 2 [Internet]. University of Bristol. 2019.

9 UK Biobank [Internet]. 2017. Available from: http://www.nealelab.is/blog/2017/7/19/rapid-gwas-of-thousands-of-phenotypes-for-337000-samples-in-the-uk-biobank. Accessed 10 May 2021.
